# Supplementary material for: Smooth Interpolating Curves with Local Control and Monotone Alternating Curvature
Source: Comput Graph Forum. 2022 Oct 6;41(5):25–38. doi: 10.1111/cgf.14600 (PMC9827861; doi:10.1111/cgf.14600)
Supplement: Supplementary file 1 — Supplement Material [file CGF-41-25-s001.zip › Local-Smooth-Interpolating-MonoCurvature/extern/clothoids/submodules/quarticRootsFlocke/docs/api-cpp/class_a00027.html]

Class Quadratic — Quartic Roots v1.0.0

### Navigation

- index
- toc
- next
- previous
- Quartic Roots »
- C++ API »
- Class Quadratic

# Class Quadratic¶

- Defined in File PolynomialRoots.hh

## Class Documentation¶

class PolynomialRoots::Quadratic¶
:   Quadratic polynomial class

    **Constructor**

    ```
    double a = 1;
    double b = 2;
    double c = 3;
    Quadratic q(a,b,c); // build an solve `a x^2 + b x + c = 0`

    Quadratic q;
    q.setup(a,b,c); // build an solve `a x^2 + b x + c = 0`
    ```

    **Get kind of solution**

    ```
    int  nroots            = q.numRoots(); 
    bool has_complex_roots = q.complexRoots();
    bool has_a_double_root = q.doubleRoot();
    ```

    **Get real roots**

    ```
    double r_min = 0;
    double r_max = 2;
    double r[2];
    int nroots;
    nroots = p.getRealRoots( r );
    nroots = p.getPositiveRoots( r );
    nroots = p.getNegativeRoots( r );
    nroots = p.getRootsInRange( r_min, r_max, r );
    nroots = p.getRootsInOpenRange( r_min, r_max, r );
    ```

    **Get roots**

    ```
    double r0 = p.real_root0(); 
    double r1 = p.real_root1();
    complexType r0 = p.root0();
    complexType r1 = p.root1();

    complexType r;
    double re, im;
    p.getRoot0( re, im );
    p.getRoot0( r );
    p.getRoot1( re, im );
    p.getRoot1( r );
    ```

    **Evaluate polynomial**

    ```
    {double or complex} v, x; 
    v = p.eval( x );

    p.eval( x, p, dp );
    ```

    **Information**

    ```
    p.info( cout );
    bool ok = p.check( cout );
    ```

    Public Functions

    inline Quadratic()¶

    inline Quadratic(valueType a, valueType b, valueType c)¶
    :   Build the object that store the roots of the quadratic polynomial

        \[ q(x) = a x^2 + b x + c \]

        Parameters
        :   - **a** – **[in]** leading coefficient of \( q(x) \)
            - **b** – **[in]** coefficient of \( x \)
            - **c** – **[in]** coefficient of \( x^0 \)

    inline void setup(valueType a, valueType b, valueType c)¶
    :   Setup the object that store the roots of the quadratic polynomial

        \[ q(x) = a x^2 + b x + c \]

        Parameters
        :   - **a** – **[in]** leading coefficient of \( q(x) \)
            - **b** – **[in]** coefficient of \( x \)
            - **c** – **[in]** coefficient of \( x^0 \)

    inline indexType numRoots() const¶
    :   Return the number of computed roots of

        \[ q(x) = a x^2 + b x + c \]

        Normally return 2 but if e.g. \( a = 0 \) return 1 or less depending on the values of \( a, b, c \)

        Returns
        :   number of computed roots

    inline bool complexRoots() const¶
    :   true if roots are complex conjugated

    inline bool doubleRoot() const¶
    :   true if \( p(x) = a x^2 + b x + c = (x-r)^2 \)

    indexType getRealRoots(valueType r[]) const¶
    :   Get the real roots

        Parameters
        :   **r** – **[out]** vector that will be filled with the real roots

        Returns
        :   the total number of real roots, 0, 1 or 2

    indexType getPositiveRoots(valueType r[]) const¶
    :   Get positive real roots

        Parameters
        :   **r** – **[out]** vector that will be filled with the real roots

        Returns
        :   the total number of positive real roots, 0, 1 or 2

    indexType getNegativeRoots(valueType r[]) const¶
    :   Get negative real roots

        Parameters
        :   **r** – **[out]** vector that will be filled with the real roots

        Returns
        :   the total number of negative real roots, 0, 1 or 2

    indexType getRootsInRange(valueType a, valueType b, valueType r[]) const¶
    :   Get real roots in a closed range

        Parameters
        :   - **a** – **[in]** left side of the range
            - **b** – **[in]** right side of the range
            - **r** – **[out]** vector that will be filled with the real roots

        Returns
        :   the total number of real roots in the range \( [a,b] \)

    indexType getRootsInOpenRange(valueType a, valueType b, valueType r[]) const¶
    :   Get real roots in an open range

        Parameters
        :   - **a** – **[in]** left side of the range
            - **b** – **[in]** right side of the range
            - **r** – **[out]** vector that will be filled with the real roots

        Returns
        :   the total number of real roots in the open range \( (a,b) \)

    inline valueType real\_root0() const¶
    :   The first real root

    inline valueType real\_root1() const¶
    :   The second real root

    inline complexType root0() const¶
    :   The first complex root

    inline complexType root1() const¶
    :   The second complex root

    inline void getRoot0(valueType &re, valueType &im) const¶
    :   Get the first root (complex or real)

        Parameters
        :   - **re** – **[out]** the first complex root, real part
            - **im** – **[out]** the first complex root, imaginary part

    inline void getRoot0(complexType &r) const¶
    :   Get the first root (complex or real)

        Parameters
        :   **r** – **[out]** the first complex root

    inline void getRoot1(valueType &re, valueType &im) const¶
    :   Get the second root (complex or real)

        Parameters
        :   - **re** – **[out]** the second complex root, real part
            - **im** – **[out]** the second complex root, imaginary part

    inline void getRoot1(complexType &r) const¶
    :   Get the second root (complex or real)

        Parameters
        :   **r** – **[out]** the second complex root

    inline valueType eval(valueType x) const¶
    :   Evaluate the quadratic polynomial

        Parameters
        :   **x** – value where compute \( p(x) \)

        Returns
        :   the value \( p(x) \)

    inline complexType eval(complexType const &x) const¶
    :   Evalute the quadratic polynomial

        Parameters
        :   **x** – **[in]** value where compute \( p(x)=ax^2+bx+c \)

        Returns
        :   the value \( p(x) \)

    void eval(valueType x, valueType &p, valueType &dp) const¶
    :   Evaluate the polynomial with its derivative

        Parameters
        :   - **x** – **[in]** value where compute \( p(x) \)
            - **p** – **[out]** value \( p(x) \)
            - **dp** – **[out]** value \( p'(x) \)

    void info(std::ostream &s) const¶
    :   Print info of the roots of the polynomial.

    bool check(std::ostream &s) const¶
    :   Check tolerance and quality of the computed roots

### Quick search

### Table of Contents

- Quartic Roots Solver
- C++ API

«
hide menu

menu
sidebar
»

### Navigation

- index
- toc
- next
- previous
- Quartic Roots »
- C++ API »
- Class Quadratic

© Copyright 2021, Enrico Bertolazzi.
Created using Sphinx 4.2.0.
